# The regulator of calcineurin 1 increases adenine nucleotide translocator 1 and leads to mitochondrial dysfunctions

**DOI:** 10.1111/jnc.13900

**Published:** 2016-12-20

**Authors:** Hui Jiang, Chen Zhang, Yu Tang, Juan Zhao, Tan Wang, Heng Liu, Xiulian Sun

**Affiliations:** ^1^ Otolaryngology Key LabQilu Hospital of Shandong UniversityJinanShandongChina; ^2^ Department of Pediatrics2nd Hospital of Shandong UniversityJinanShandongChina; ^3^ Department of GeriatricsQilu Hospital of Shandong UniversityJinanShandongChina; ^4^ Brain Research InstituteQilu Hospital of Shandong UniversityJinanShandongChina

**Keywords:** Alzheimer's disease, ANT1, mitochondria dysfunction, neuronal apoptosis, RCAN1.1

## Abstract

The over‐expression of regulator of calcineurin 1 isoform 1 (RCAN1.1) has been implicated in mitochondrial dysfunctions of Alzheimer's disease; however, the mechanism linking *RCAN1.1* over‐expression and the mitochondrial dysfunctions remains unknown. In this study, we use human neuroblastoma SH‐SY5Y cells stably expressing RCAN1.1S and rat primary neurons infected with RCAN1.1S expression lentivirus to study the association of RCAN1 with mitochondrial functions. Our study here showed that the over‐expression of RCAN1.1S remarkably up‐regulates the expression of adenine nucleotide translocator (ANT1) by stabilizing *ANT1* mRNA. The increased ANT1 level leads to accelerated ATP–ADP exchange rate, more Ca^2+^‐induced mitochondrial permeability transition pore opening, increased cytochrome *c* release, and eventually cell apoptosis. Furthermore, knockdown of *ANT1* expression brings these mitochondria perturbations caused by RCAN1.1S back to normal. The effect of RCAN1.1S on ANT1 was independent of its inhibition on calcineurin. This study elucidated a novel function of RCAN1 in mitochondria and provides a molecular basis for the RCAN1.1S over‐expression‐induced mitochondrial dysfunctions and neuronal apoptosis.

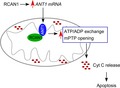

**Abbreviations used**
AD: Alzheimer's disease
ANT: adenine nucleotide translocator
BCL‐2: B‐cell lymphoma‐2
BKA: bongkrekic acid
CATR: carboxyatractyloside
COX IV: cytochrome *c* oxidase subunit IV
Cyt *c*: cytochrome *c*
mPTP: mitochondrial permeability transition pore
RCAN1: regulator of calcineurin 1
ROS: reactive oxygen species
SOD2: superoxide dismutase 2

Mitochondria are the critical regulators of neuronal death which is a key mark in neurodegeneration. And dysfunctions of mitochondria have been suggested in aging and neurodegenerative diseases including Alzheimer's disease (AD) and Parkinson's disease (Lin and Beal 2006). Mitochondrial dysfunctions are one of the most early and prominent features in vulnerable neurons in neurodegenerative diseases, including impaired mitochondrial respiration, increased reactive oxygen species generation, mitochondrial DNA damage, decreased mitochondrial mass, and abnormal mitochondrial dynamics (de la Monte *et al*. 2000; Zhu *et al*. 2006; Querfurth and LaFerla 2010; Swerdlow *et al*. 2010). Adenine nucleotide translocator 1 (ANT1), or the ADP/ATP translocator 1, is the most abundant protein in the inner mitochondrial membrane. It forms as a homodimer, a gated channel by which ADP is brought into and ATP brought out of the mitochondrial matrix. In addition to the translocase activity, ANT has regulatory role in mitochondrial permeability transition pore (mPTP) function and is involved in mitochondria‐mediated apoptosis (Kokoszka *et al*. 2004; Sharer 2005; Dahout‐Gonzalez *et al*. 2006). mPTP, a non‐specific pore in the mitochondrial inner membrane, opens by the primary trigger of elevated matrix Ca^**2+**^, leading to permeability to any molecule of < 1.5 kDa (Halestrap *et al*. 2002). As the result of this pore opening, the mitochondrial electrochemical hydrogen ion gradient dissipates, the matrix is depleted of pyridine nucleotides, mitochondria swell because of the osmotic uptake of water, cytochrome *c* is released into cytosol, and eventually leads to cell apoptosis (Soane *et al*. 2007). mPTP opening and energy crisis have been considered to play important roles in acute and chronic neurodegeneration. There are two conformations of ANT1, matrix conformation (m‐conformation) and cytosol conformation (c‐conformation), which could be induced and stabilized by specific ligands bongkrekic acid (BKA) and carboxyatractyloside (CATR), respectively (Dahout‐Gonzalez *et al*. 2006). Regulations of mPTP as well as the translocase activity of ANT1 likely lie on the conformational grounds, indicated by the fact that BKA can inhibit mPTP opening while CATR can facilitate it, and both the two ligands can inhibit ATP–ADP exchange (Dahout‐Gonzalez *et al*. 2006). Many studies have demonstrated that appropriate ANT1 level was vital to mitochondrial function and cell survival (Sharer 2005; Kawamata *et al*. 2011; Liu and Chen 2013), implying that the expression of *ANT1* must be tightly controlled to avoid any deleterious effect.

Regulator of calcineurin 1 (*RCAN1*), also known as *MCIP1*,* DSCR1*,* adapt78*, and calcipressin, is located at 21q22.12 and consists of seven exons and six introns. Alternatively, splicing the first four exons generates four isoforms of RCAN1 that differ only in their N‐terminal (Fuentes *et al*. 1997). Different usage of two translational start codon (AUG) resulted in two isoforms of RCAN1.1, RCAN1.1L and RCAN1.1S with 55 amino acids longer in RCAN1.1L (Wu and Song 2013). RCAN1.1L is the predominant form expressed in the brain. RCAN1.1S and RCAN1.4, differing in 28 amino acids in N‐terminus, have tissue‐specific expression pattern by usage of two alternative promoters (Sun *et al*. 2014b). RCAN1.1 isoform is primarily abundant in the fetal and adult brains. Previous data have shown that RCAN1.1 expression is elevated in the cortex of AD patients and the over‐expression may contribute to AD pathogenesis (Ermak *et al*. 2001; Ermak and Davies 2013). We recently report that the degradation of RCAN1.1 is mediated by both chaperon‐mediated autophagy and ubiquitin proteasome pathways (Liu *et al*. 2009); RCAN1.1 is elevated in the brains of AD and DS patients and RCAN1 over‐expression facilitates neuronal apoptosis through caspase 3 activation (Sun *et al*. 2011); the transcription of *RCAN1.4* can be activated by NF‐κB (Zheng *et al*. 2014) and RCAN1.4 over‐expression exacerbates calcium overloading‐induced neuronal apoptosis (Sun *et al*. 2014b). Our recent paper also showed that RCAN1.1S and RCAN1.4 inhibited NF‐κB and suppressed lymphoma growth that is independent of its inhibition on calcineurin (Liu *et al*. 2015). Our data also showed that RCAN1 was located in ER and promoted N‐glycosylation via oligosaccharyltransferase (Wang *et al*. 2014). These studies suggested that RCAN1 is a multifunctional protein. The RCAN1.1‐related mitochondrial dysfunctions include reduction of mitochondrial mass, decline of cellular ATP level, opening of mPTP, and activation of caspase signal pathway (Chang and Min 2005; Sun *et al*. 2011, 2014a,b; Ermak *et al*. 2012), but the underlying molecular basis remains to be discovered. A report from *Drosophila* species proposes that nebula (*Drosophila* homolog of RCAN1) can interact with ANT1 to modulate mitochondrial function (Chang and Min 2005); however, whether RCAN1 interacts with ANT1 in mammalian system remains elusive.

Our study here showed that the over‐expression of RCAN1.1S remarkably elevated ANT1 expression by stabilizing *ANT1* mRNA. The increased ANT1 level led to abnormal mitochondrial functions including accelerated ATP–ADP exchange rate, more Ca^2+^‐induced mPTP opening, increased Cyt *c* release, and eventually cell apoptosis in neuronal cell lines. Furthermore, knockdown of *ANT1* expression by shRNA vector brought these mitochondrial perturbations caused by RCAN1.1S back to normal. The study here demonstrated that RCAN1 impeded mitochondrial functions through ANT1.

## Materials and methods

The experimental protocols were approved by the Animal Care and Protection Committee of Shandong University and institutional Ethics Committees of Qilu Hospital, and in compliance with ARRIVE guideline.

### Cell culture

YD2 cells were generated by stable transfection of pRCAN1.1S‐6myc into human neuroblastoma SH‐SY5Y cells as previously described (Liu *et al*. 2009). Rat primary neurons were isolated from E18 pregnant rats and cultured as previously described (Sun *et al*. 2011). Pregnant rats were bought from experimental animal center of Shandong University. All cells were maintained at 37°C in an incubator containing 5% CO_2_.

### Plasmids construction and transfection

The *RCAN1.1S* expression plasmid pcDNA3.1‐RCAN1.1S‐6myc and the *RCAN1* knockdown plasmid pSuper‐siRCAN1 were generated as previously described (Liu *et al*. 2009). RCAN1.1S refers to the shorter isoform of NCBI seq NM_004414. RCAN1.4 refers to NCBI seq NM_203418. The truncation plasmids pcDNA3.1‐RCAN1.1S 1‐103‐6myc and pcDNA3.1‐RCAN1.1S 141‐197‐6myc were constructed as described previously (Liu *et al*. 2015). The cDNA of *ANT1* was PCR amplified with ANT1‐1F (5’‐CCGGGATCCTTCGACATATTTTTTGAT) and ANT1‐894R (5’‐CCGGATATCTGCAGAATTCGCCACC) and cloned into p3 × flagCMV10 vector to generate *ANT1* expression plasmid p3 × flagCMV10‐ANT1. The two strands of siANT1‐147F (5’‐GATCCCCGCAGTACAAAGGGATCATTGATTCAAGAGATCAATGATCCCTTTGTACTGC) and siANT1‐147R (5’‐AGCTTAAAAAGCAGTACAAAGGGATCATTGATCTCTTGAATCAATGATCCCTTTGTACTGC) were annealed and ligated into pSuper vector to generate the *ANT1* knockdown plasmid pSuper‐siANT1. All transfections were carried out with Lipofectamine™ 2000 (Thermo Fisher Scientific) transfection reagent according to the manufacturer's instructions.

### Lentivirus construction and Infection

The cDNA of *RCAN1.1S* and *ANT1* were PCR amplified and cloned into lentivirus vector pWPXL. The lentivirus expression vector was co‐transfected with psPAX2 and pMD2.G into HEK293T cells for lentivirus production. Lentivirus was harvested from the culture media 48 h after transfection and precipitated with PEG8000. The titer of lentivirus produced is about 10^7^ pfu/mL. Rat primary neurons were infected with a MOI (multiplicity of infection) of 5 for 8 h at 37°C, followed by replacement of the infection media with conditioned culture media and incubated for 3 days. About 30–50% of neurons were transduced by visualizing GFP (green fluorescence protein) expression that was fused with *RCAN1.1S* or *ANT1*.

### Immunoblotting analysis

For immunoblotting analysis, cells or isolated mitochondria were lysed in radio‐immunoprecipitation assay buffer (1% Triton X100, 1% sodium deoxycholate, 4% sodium dodecyl sulfate, 0.15 M NaCl, 0.05 M Tris‐HCl, pH 7.2) supplemented with protease inhibitors. The lysates were resolved by sodium dodecyl sulfate–polyacrylamide gel electrophoresis and the immunoblotting was performed as described previously (Liu *et al*. 2009). The primary antibodies used were mouse 9E10 mAb, M2 mAb, anti‐ANT1 mAb, anti‐superoxide dismutase 2 (SOD2) mAb, and anti‐β‐actin mAb. The endogenous RCAN1.1 antibody DCT3 (a polyclonal rabbit against the last 20 aa in the RCAN1 C‐terminus) was used to detect endogenous expression of RCAN1 (Sun *et al*. 2011). Detection and quantifications were performed with the Li‐Cor Odyssey imaging system and its software (Liu *et al*. 2009).

### Mitochondrial isolation

All steps were carried out at 0–4°C; mitochondrial isolation from YD2 and SH‐SY5Y cells was performed as described previously with minor modifications (Sun *et al*. 2011). The cell homogenate was centrifuged twice at 800 *g* for 5 min in order to get rid of the nuclei and remaining intact cells; the supernatant was centrifuged at 8000 *g* for 15 min to pellet crude mitochondria. The supernatant was transferred to a new tube and centrifuged for an additional time in order to remove any remaining mitochondria, and collected as the cytosol fraction. The crude mitochondria were layered over a 1.0/1.5 M discontinuous sucrose gradient containing protease and phosphatase inhibitors and centrifuged at 100 000 *g* (Beckman Optima MAX‐XP Ultracentrifuge MLS 50 rotor (Beckman Coulter, Krefeld, Germany)) for 1 h. The purified mitochondria were collected from the 1.0 to 1.5 M sucrose interface by pipetting. 9E10 mAb was used to detect exogenous myc‐tagged RCAN1.1S of YD2 cells, and DCT3 to detect endogenous RCAN1.1 of SH‐SY5Y cells. Several mitochondrial markers were used to quantify mitochondria: B‐cell lymphoma‐2 (BCL‐2) for the outer membrane, Cyt *c* for the intermembrane space, SOD2 for matrix, and Cyt *c* oxidase subunit IV (COX IV) for the inner membrane. In the mPTP‐related assays, metabolically active mitochondrial isolation from cultured rat primary neurons was performed according to the method previously described (Almeida and Medina 1998).

### RT‐PCR

RT‐PCR was performed as previously described (Liu *et al*. 2009). Specific primers used to amplify *RCAN1.1S* gene are RCAN1.1F (5’‐CGACTGGAGCTTCATTGACT) and RCAN1.1R (5’‐CCCAAGCTTTCCGCTGAGGTGGATCGGCGTGTA). Specific primers used to amplify a 125‐bp fragment of *ANT1* gene are ANT1‐202F (5’‐CTCTCCTTCTGGAGGGGTAAC) and ANT1‐327R (5’‐GAACTGCTTATGCCGATCCAC). A 141‐bp fragment of human β*‐*actin amplified with primers actin‐F (5’‐GACAGGATGCAGAAGGAGATTACT) and actin‐R (5’‐TGATCCACATCTGCTGGAAGGT) was used as internal control. To verify that the read‐out of RT‐PCR results was linear, different amplification cycle numbers of *RCAN1.1S*,* ANT1*, and β*‐actin* genes were performed. Samples were analyzed on 1.5% agarose gel. Image J software was used to quantify and analyze the data. Mitochondrial DNA (mtDNA) was determined to evaluate the mitochondrial content using quantitative real‐time PCR as previously described (Bai and Wong 2005). *RPPH1* gene was used as nuclear gene normalizers for the mtDNA content (TaqMan Copy Number Reference Assay from ABI (Waltham, MA, USA).

### Degradation of *ANT1* mRNA and protein

For the measuring of *ANT1* mRNA degradation, SH‐SY5Y and YD2 cells were treated with 1 μg/mL actinomycin D (Act D) to inhibit de novo RNA synthesis for 0, 3, 6, 9, and 12 h. *ANT1* mRNA level was measured by RT‐PCR: isolated RNA was treated with recombinant DNase I before reverse transcription to prevent contamination of genomic DNA, random primer (3801; Takara, Dalian, China) was used to synthesize the first strand of cDNA, and 18s rRNA was used as an internal control. The specific primers amplifying a 232‐bp fragment of 18s gene were 18srRNA‐F1464 (5’‐CAGCCACCCGAGATTGAGCA) and 18s rRNA‐R1696 (5’‐TAGTAGCGACGGGCGGTGTG). The primers amplifying a 140‐bp fragment of *Dynamin* 1 (*DNM1*) gene were DNM1‐F237 (5’‐ATATGCCGAGTTCCTGCACTG) and DNM1‐R376 (5’‐AGTAGACGCGGAGGTTGATAG). For the measuring of ANT1 protein degradation, SH‐SY5Y cells were co‐transfected with pcDNA3.1‐RCAN1.1‐6myc and p3 × flagCMV10‐ANT1; 48 h after transfection, cells were treated with 100 μg/mL cycloheximide (CHX) which could interfere with the translocation step to inhibit protein synthesis for 0, 12, 24, and 36 h, respectively; the level of ANT1 protein was measured by western blot and detected by M2 mAb.

### ATP–ADP exchange rate assay

ATP–ADP exchange rate solely mediated by ANT was measured according to a method developed by Kawamata *et al*. (2010) by exploiting the differential affinity of ADP and ATP to Mg^2+^. A total quantity of 40 μg/mL digitonin was used to permeabilize cells; 2 mM ADP was added to start the mitochondrial phosphorylation; magnesium green fluorescence was recorded in, calibrated, and converted to [ATP]t appearing in the reaction medium using standard binding equations listed in the published method (Chinopoulos *et al*. 2009; Kawamata *et al*. 2010). The fitted slope obtained by the linear regression of this time course of [ATP]t appearing in the reaction medium reflects ATP–ADP exchange rate mediated by the ANT. The ATP–ADP exchange rate mediated by the ANT was validated in this assay by sequentially adding 4 nM CATR to the reaction medium, which resulted in a complete halt of ATP rise in the media after three additions.

### Determination of mPTP opening

mPTP opening was determined as previously described using the calcein‐CoCl_2_ bleaching assay (Petronilli *et al*. 1999). Briefly, cultured cells (grown in wells of a 24‐well plate) were washed in reaction buffer (140 mM NaCl, 5.0 mM KCl, 10 mM HEPES, 2.0 mM CaCl_2_, 1.0 mM MgCl_2_, and 10 mM glucose, pH 7.4), and pre‐incubated in fresh buffer A containing 2 μM fluorescence dye calcein‐AM and 1 mM CoCl_2_ at 37°C for 30 min, then washed three times with buffer A, and the initial calcein fluorescent signals was recorded by Varioskan flash instruments (Thermo Scientific, Shanghai, China) at 25°C with the excitation wavelength of 494 nm and emission wavelength of 517 nm. mPTP opening was induced by adding 500 μM CaCl_2_ along with the 5 μM Ca^2+^ ionophore ionomycin in the presence or absence of ANT1 ligand, and the fluorescence signals were measured again. The added ANT1 ligand was 1 μM carboxyatractyloside which sensitized the mPTP to Ca^**2+**^, or 5 μM BKA which made the mPTP insensitive to Ca^**2+**^. The protein concentration for each well was measured by the Bradford protein assay. The fluorescent signals were normalized to total protein content. The decreased percentage of initial fluorescent signals could be interpreted as mPTP opening.

### Swelling of energized mitochondria

The Ca^2+^‐triggered mitochondrial swelling assay was performed as follows: isolated mitochondria (0.4 mg/mL) were incubated in KCl media (125 mM KCl, 2 mM K_2_HPO_4_, 1 mM MgCl_2_, 20 mM HEPES, 5 mM glutamate, 5 mM malate, and 2 μM rotenone, pH7.4) for 10 min in the presence or absence of ANT1 ligand (1 μM CATR or 5 μM BKA). Mitochondrial swelling was triggered by the addition of Ca^2+^ (500 nmol/mg mitochondrial protein); the mitochondrial swelling caused by an influx of solutes across the inner membrane was observed by immediately and continuously recording the decrease in absorbance at 540 nm on Varioskan flash instruments (Thermo Scientific).

### Ca^2+^ retention capacity of mitochondria

Mitochondrial Ca^2+^ retention capacity was determined under energized conditions. Isolated mitochondria (0.4 mg/mL) were suspended in 1 mL KCl media containing 0.5 μM Calcium green‐5N in the presence or absence of ANT1 ligand (1 μM CATR or 5 μM BKA). Fluorescence changes were continuously measured at 25°C with the excitation wavelength of 506 nm and emission wavelength of 531 nm. A total quantity of 4 μL aliquots of a 20 mM CaCl_2_ solution (20 mM CaCl_2_, 127 mM KCl, 1 mM MgCl_2_, 20 mM HEPES, 5 mM glutamate, 5 mM malate, pH 7.4) were added every 2 mins to introduce 200 nmol Ca^2+^/mg protein until mPTP opening indicated a double increase above the baseline reading of fluorescence. The total amount of Ca^2+^ added can be interpreted as Ca^2+^ retention capacity.

### Detection of Cyt *c* release and TUNEL staining

For detection of Cyt *c* release, mitochondria and cytosol fractions were separated as mentioned above. Western blot was used to detect Cyt *c* release from mitochondria to cytosol. SOD2 was used as mitochondrial marker and GAPDH for cytosolic marker. For TUNEL staining, cells were fixed in 4% paraformaldehyde for 40 min, permeabilized with 0.1% TritonX‐100 for 10 min, and stained with 1 μg/mL DAPI (4',6‐diamidino‐2‐phenylindole) (D9542; Sigma‐Aldrich, Shanghai, China) at 25°C for 10 min. TUNEL staining was performed using the Roche‐In Situ Cell Death Detection Kit according to the manufacturers’ instructions. Results were analyzed by fluorescence microscopy (Leica DMI4000B, Wetzlar, Germany).

### Statistical analysis

All the experiments were repeated at least three times. One representative picture is shown in figures. Quantifications were from three or more independent experiments. Data were analyzed by Student's *t*‐test. Values represent means ± SE; *p* < 0.05 was considered as statistically significant.

### Data accessibility

The original unprocessed data are available in supporting information online.

### Materials

The materials used were as follows: lipofectamine™ 2000 transfection reagent (Life Technologies, Grand Island, NY, USA), lentivirus vector pWPXL (from Addgene, Cambridge, MA, USA), protease inhibitors (Cat#: 04693116001; Roche Molecular Biochemicals, Indianapolis, IN, USA), 9E10 mAb (anti‐c‐myc; ab32; Abcam, USA), M2 mAb (anti‐flag; F1804; Sigma‐Aldrich), anti‐ANT1 mAb (ab110322; Abcam, Cambridge, MA, USA), anti‐SOD2 mAb (12656‐RP02; Sino Biological, Beijing, China), BCL‐2 (BS1511; Bioworld Technology, Nanjing, China), Cyt *c* (AC909; Beyotime, Guangzhou China), COX IV (4850; Cell Signaling Technology, Beverly, MA, USA), anti‐β‐actin mAb (AC‐15; Sigma‐Aldrich), TaqMan Copy Number Reference Assay (4403316; Life Technologies), FK506 (F4679; Sigma‐Aldrich), actinomycin D (A9415; Sigma‐Aldrich), DNAseI (2270A; Takara), CHX (S1560; Beyotime), digitonin (D141; Sigma‐Aldrich), random primer (3801; Takara), Magnesium Green (M‐3733; Life Technologies); carboxyatractyloside (C4992; Sigma‐Aldrich), calcein‐AM (17783; Sigma‐Aldrich), CoCl_2_ (V900021; Sigma‐Aldrich), ionomycin (S1672; Beyotime), Bongkrekic acid (1820‐100; Biovision, Milpitas, CA, USA), Calcium green‐5N (c‐3737; Life Technologies), and DAPI (D9542; Sigma‐Aldrich); Roche‐In Situ Cell Death Detection Kit (12156792910; Roche Molecular Biochemicals).

## Results

### RCAN1.1S increased *ANT1* mRNA and protein levels

**Figure 1:**
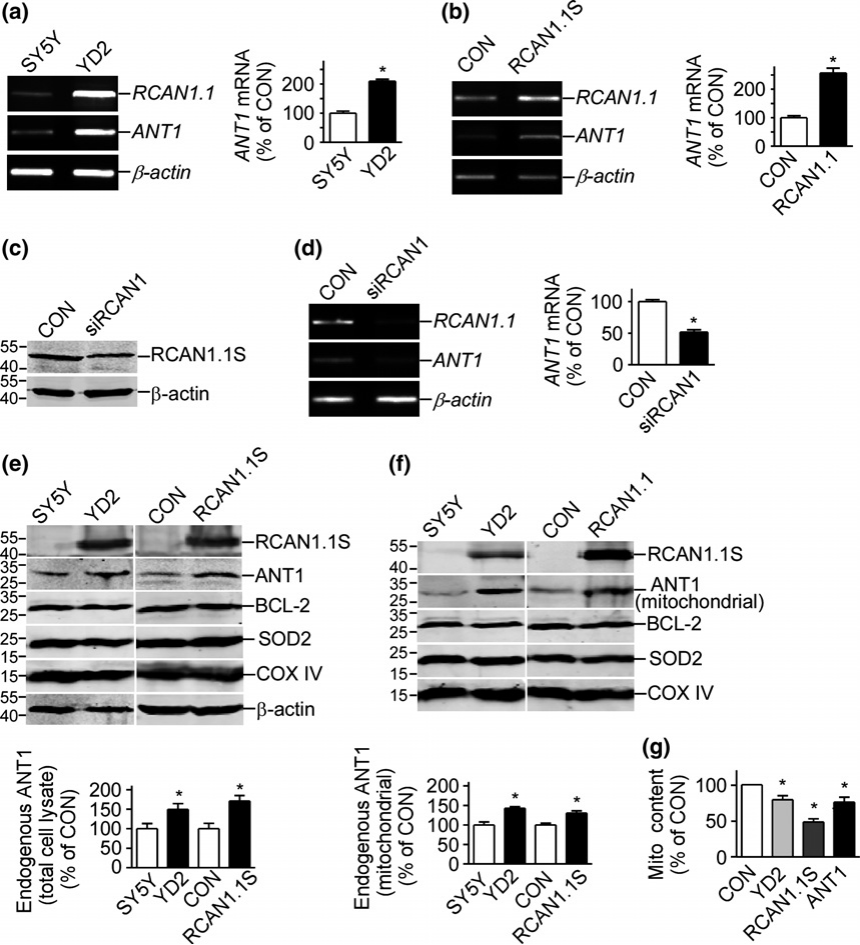
Over‐expression of *RCAN1.1S* increased adenine nucleotide translocator (*ANT1*) mRNA and protein levels. (a) RT‐PCR showed that stable over‐expression of *RCAN1.1S* in YD2 cells significantly increased *ANT1* mRNA levels. β‐actin was amplified as the internal control (row 3). (b) RT‐PCR showed that transient (48 h) over‐expression of *RCAN1.1S* in SH‐SY5Y cells also remarkably increased *ANT1* mRNA levels. (c) pSuper‐RCAN1 and pcDNA3.1‐RCAN1.1S‐6myc were co‐transfected into SH‐SY5Y cells. Western blot was used to confirm the knockdown of *RCAN1.1* by pSuper‐RCAN1 48 h after transfection. (d) RT‐PCR showed that *RCAN1* knockdown in SH‐SY5Y cells significantly decreased *ANT1* mRNA level. The knockdown effect of pSuper‐RCAN1 was also confirmed in *RCAN1.1* mRNA level. β‐actin was amplified as the internal control. (e and f) Over‐expression of *RCAN1.1S* increased ANT1 protein levels. Endogenous ANT1 protein levels of total cell lysates (e) and mitochondria (f) were detected by anti‐ANT1 mAb in YD2 cells and pcDNA3.1‐RCAN1.1S‐6myc transiently transfected SH‐SY5Y cells. Over‐expressed RCAN1.1S protein in YD2 and transfected SH‐SY5Y cells was also confirmed by 9E10 (anti‐myc) antibody. β‐actin was used as loading control for whole cell lysate and superoxide dismutase 2 (SOD2) as loading control for mitochondria fractions. Values represent mean ± SE; *n* = 4 (a–d) *n* = 5 (e and f). *p < 0.05 by Student's *t*‐test. (g) Mitochondrial DNA (mtDNA) was determined to evaluate the mitochondrial content using quantitative real‐time PCR. *RPPH1* gene was used as nuclear gene normalizers for the mtDNA content (TaqMan Copy Number Reference Assay from ABI).

To reveal the mechanism of RCAN1‐ANT1 interaction, we examined the expression of *ANT1* in YD2 cells and SH‐SY5Y cells transiently over‐expressing RCAN1.1S. RT‐PCR was used to examine the *ANT1* mRNA expression. RT‐PCR showed that *ANT1* mRNA level in YD2 cells was significantly increased to 209.60 ± 6.94% of control cells (*p* < 0.01, Fig. 1a). *ANT1* mRNA expression was also elevated to 256.80 ± 17.45% of control in SH‐SY5Y cells transiently transfected with *RCAN1.1* expression plasmid (*p* < 0.01, Fig. 1b). Furthermore, *ANT1* mRNA was decreased to 51.79 ± 3.47% of control in cells with RCAN1 knocked down (*p* < 0.01, Fig. 1d). RCAN1 knockdown effect was also verified by western blot in Fig. 1(c). To further examine whether the increase of mRNA resulted in the increase of its protein expression, western blot assay was performed and showed that the overall levels of endogenous ANT1 (Fig. 1e) as well as the mitochondrial ANT1 (Fig. 1f) were increased by RCAN1.1S over‐expression. The results showed that endogenous ANT1 protein in total cell lysates was increased to 149.70 ± 14.80% in YD2 cells (*p* < 0.05, Fig. 1e, lane 2), and 179.90 ± 14.14% of control in *RCAN1.1S* transiently transfected SH‐SY5Y cells (*p* < 0.01, Fig. 1e, lane 4). Endogenous ANT1 protein in mitochondria was also increased to 142.50 ± 3.82% in YD2 cells (*p* < 0.01, Fig. 1f, lane 2), and 130.30 ± 5.80% in the *RCAN1.1* transiently transfected SH‐SY5Y cells (*p* < 0.01, Fig. 1f, lane 4). To confirm that the RCAN1.1S effect was specific for ANT1, we also detected the level of COX IV which was also located at mitochondrial inner membrane and BCL‐2 which could interact with ANT1 (Brenner *et al*. 2000) in total cell lysates and mitochondrial fractions (Fig. 1e and f). Quantitative results showed that the levels of COX IV and BCL‐2 were not altered by the over‐expressed RCAN1.1S (*p* > 0.05; quantitative data in supporting information). In addition, mitochondrial content was associated with the level of mitochondrial protein, so we evaluated mitochondrial content by mtDNA copy number; the results demonstrated that the mtDNA content determined by normalized DLOOP copy number significantly decreased to 79.20 ± 11.16% in YD2, to 48.48 ± 8.84% in RCAN1.1S transiently transfected SH‐SY5Y cells, and to 75.80 ± 14.07% in ANT1 transiently transfected SH‐SY5Y cells (Fig. 1g). These data clearly indicated that *ANT1* expression was up‐regulated by RCAN1.1S at both the mRNA and protein levels.

### RCAN1.1S retarded the degradation rate of *ANT1* mRNA

**Figure 2:**
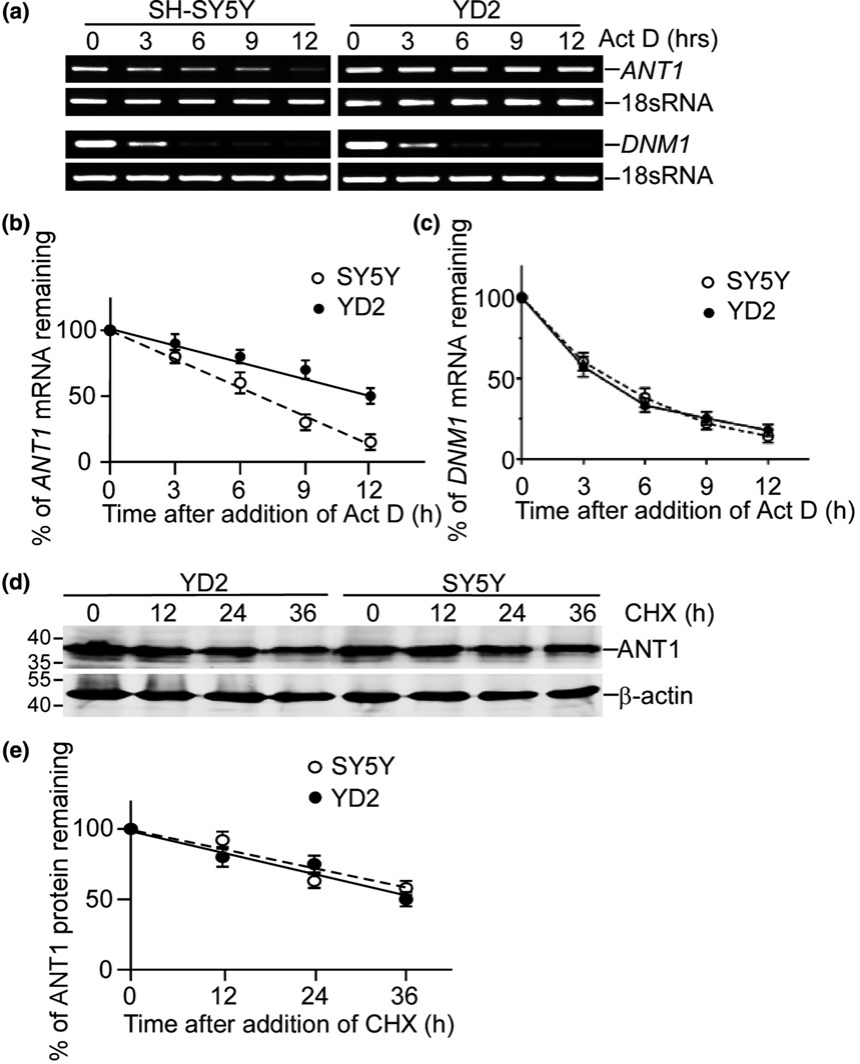
Over‐expression of *RCAN1.1S* stabilized adenine nucleotide translocator (*ANT1*) mRNA (a). SH‐SY5Y and YD2 cells were treated with 1 μg/mL actinomycin D (Act D) for 0, 3, 6, 9, and 12 h. RT‐PCR was used to detect the *ANT1* mRNA *and dynamin 1* (*DNM1*) mRNA. 18S rRNA was amplified as an internal control. (b). Quantification of *ANT1* mRNA in (a). (c). Quantification of *DNM1* mRNA in (a). Values represent mean ± SE; *n* = 5. mRNA and protein levels at 0 h were artificially set to 100%. (d). *ANT1* expression plasmid p3 × flagCMV10‐ANT1 was transfected in SH‐SY5Y and YD2 cells. A total quantity of 100 μg/mL cycloheximide (CHX) was added 48 h after transfection and cells were collected at 0, 12, 24, 36 h after treatment. Western blot was used to detect the ANT1 protein level using M2 mAb and β‐actin was used as loading control. (e). Quantification of (d). Values represent mean ± SE; *n* = 5. mRNA and protein levels at 0 h were artificially set to 100%.

To further elucidate the molecular mechanism of the ANT1 mRNA up‐regulation by RCAN1, the degradation rate of *ANT1* mRNA was measured by actinomycin D (Act D) chase assay. 18s rRNA was chosen as an internal control (Selvey *et al*. 2001). The data showed that *ANT1* mRNA was more stable in YD2 cells compared with SH‐SY5Y cells (*p* < 0.05 starting from 3 h point, Fig. 2a and b). To confirm that the increased stability is specific to the *ANT1* transcript rather than a change in mRNA stability throughout the cell, we also detected the degradation of *DNM1* mRNA whose half‐life was approximately 3 h in Act D treated SH‐SY5Y and YD2 cells. The results indicated that the stability of *DNM1* mRNA was not altered by RCAN1.1 (*p* > 0.05; Fig. 2a and c), demonstrating that the increased stability is specific to the *ANT1* transcript. CHX chase assay was used to measure the degradation rate of ANT1 protein. The data showed that there were no significant difference in the ANT1 protein stability between RCAN1.1S over‐expressed cells and control cells (*p* > 0.05, Fig. 2d and e), implying *RCAN1.1S* over‐expression did not interfere with the degradation of ANT1 protein. The data here supported that RCAN1 up‐regulated *ANT1* expression by stabilizing the *ANT1* mRNA.

### RCAN1 increased ANT1 independent of its inhibition on calcineurin

**Figure 3:**
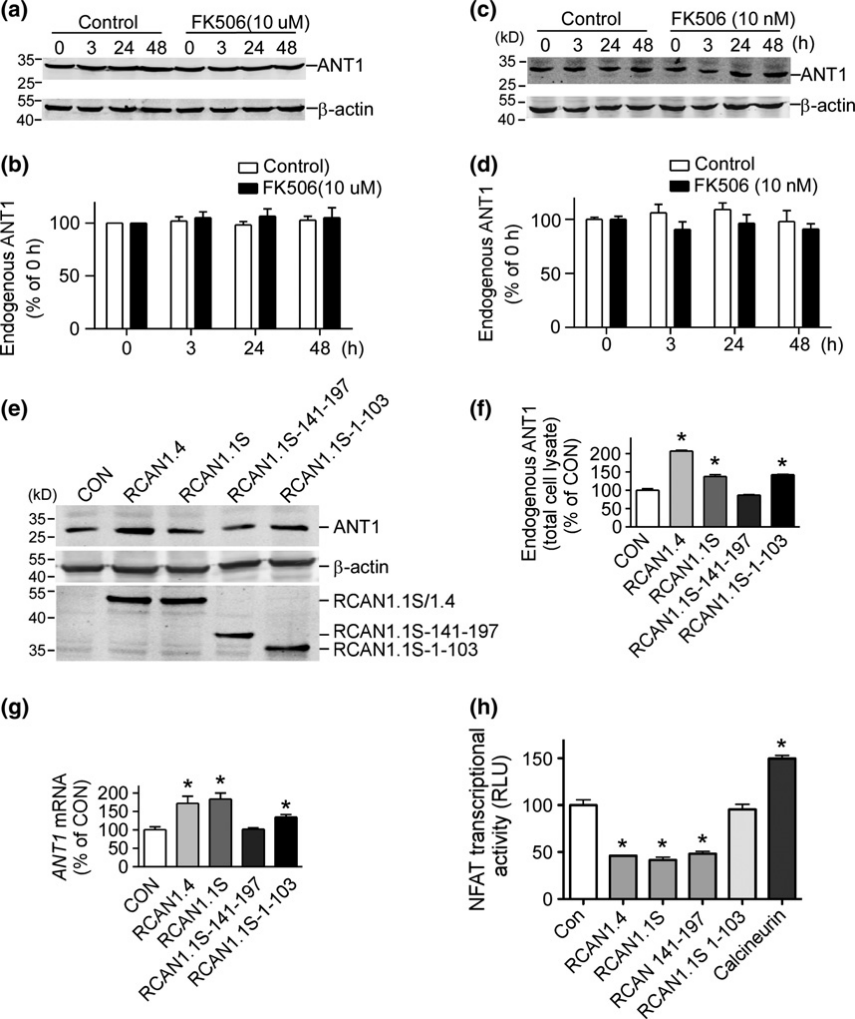
RCAN1 increased adenine nucleotide translocator (ANT1) independent of its inhibition on calcineurin. (a and c). SH‐SY5Y cells were treated with DMSO (control) and 10 μM FK506 (a) and 10 nM FK506 (c) (dissolved in DMSO, FK506+) for 0, 3, 24, and 48 h. Endogenous ANT1 level was detected in whole cell lysate. (b). Quantification of (a). (d) Quantification of (c). (e) SH‐SY5Y cells were transfected with pcDNA3.1‐RCAN1.4‐6myc, pcDNA3.1‐RCAN1.1S‐6myc, pcDNA3.1‐RCAN1.1S 1‐103‐6myc, pcDNA3.1‐RCAN1.1S 141‐197‐6myc. Forty‐eight hours after transfection, the mitochondria were isolated and detected with anti‐ANT1 antibody. RCAN1 isoforms and truncation forms were detected with anti‐myc antibody in the whole cell lysate and β‐actin was used as loading control. (f) Quantification of (e). Values represent mean ± SE; *n* = 3. mRNA and protein levels at con were artificially set to 100%. (g) SH‐SY5Y cells were transfected with pcDNA3.1‐RCAN1.4‐6myc, pcDNA3.1‐RCAN1.1S‐6myc, pcDNA3.1‐RCAN1.1S 1‐103‐6myc, pcDNA3.1‐RCAN1.1S 141‐197‐6myc. Forty‐eight hours after transfection, the total RNA was isolated and real‐time PCR was used to detect ANT1 mRNA level. (h) HEK293 cells were transfected with pNFATluc and pcDNA3.1‐RCAN1.4‐6myc, pcDNA3.1‐RCAN1.1S‐6myc, pcDNA3.1‐RCAN1.1S 1‐103‐6myc, pcDNA3.1‐RCAN1.1S 141‐197‐6myc, and calcineurin expression vector. Dual luciferase assay was used to measure the luciferase activity to reflect the NFAT transcriptional activity. Values represent mean ± SE; *n* = 3. *p < 0.05 by Student's *t*‐test.

RCAN1 can inhibit calcineurin via its C‐terminus 141–197 aa (Chan *et al*. 2005; Martinez‐Martinez *et al*. 2009). To further investigate whether the effect of RCAN1.1 over‐expression on ANT1 was associated with its inhibition on calcineurin, endogenous ANT1 was monitored in SH‐SY5Y cells treated with FK506, a pharmacological inhibitor of calcineurin. The FK506 effect was assessed both as chronic administration (24 and 48 h) and acutely (3 h) to distinguish between long‐term effects and short‐term effects. A higher dosage of 10 μM (Fig. 3a and b) and lower dosage of 10 nM (Fig. 3c and d) were used for FK506 treatment. The results showed that FK506 did not alter ANT1 level, no matter at chronic or acute administration (*p* > 0.05; Fig. 3a–d), demonstrating that the effect of RCAN1.1 on ANT1 is independent of calcineurin. The C‐terminus 141–197 aa was sufficient for inhibition of calcineurin. To further verify that RCAN1's effect is independent of its effect on calcineurin, the two isoforms of RCAN1.1S and RCAN1.4 as well as two truncations RCAN1.1S 1–103 aa and 141–197 aa were transfected into SH‐SY5Y cells. ANT1 mRNA and protein expression were assayed by RT‐PCR and western blot. The results showed that RCAN1.4, RCAN1.1S, and RCAN1.1S 1–103 increased ANT1 protein level (Fig. 3e and f) and mRNA level (Fig. 3g). The C‐terminus 141–197 aa can inhibit calcineurin‐NFAT signaling while it had no effect on ANT1 expression (lane 4 of Fig. 3e–h), indicating inhibition of calcineurin is not sufficient to increase ANT1. The N‐terminus 1–103 aa increased ANT1 expression (lane 5 of Fig. 3e–h) while having no effect on calcineurin, indicating that N‐terminus domain is sufficient for its effect on ANT1. The larger isoform 1 of RCAN1.1L also increased ANT1 expression (supplementary Fig. 1), implying that the effect of RCAN1 on ANT1 is a common effect among RCAN1 variants. These experiments demonstrated that RCAN1's effect on ANT1 is independent of its effect on calcineurin.

### RCAN1.1S accelerated ATP–ADP exchange rate via ANT1

**Figure 4:**
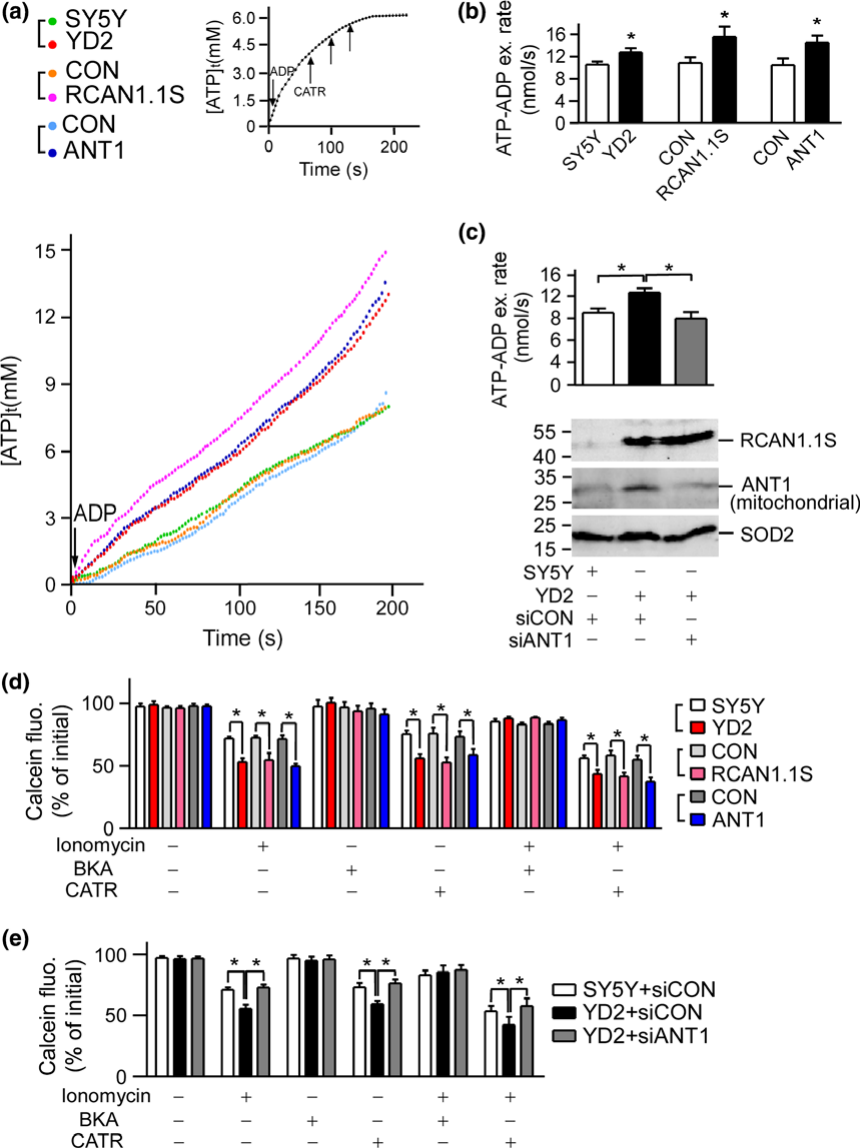
Over‐expression of *RCAN1.1S* resulted in accelerated ATP–ADP exchange rate and more Ca^2+^‐induced mitochondrial permeability transition pore (mPTP) opening. (a). Over‐expression of *RCAN1.1S* and adenine nucleotide translocator (*ANT1*) resulted in accelerated ATP–ADP exchange rate. The representative time course of [ATP]t in SH‐SY5Y, YD2, SH‐SY5Y transfected with pcDNA3.1‐RCAN1.1S‐6myc (RCAN1.1S), p3 × flagCMV10‐ANT1 (ANT1), and their empty vectors (CON). The [ATP]t was calculated from the change of the magnesium green fluorescence according to Methods. Thumbnail figure at top right confirmed the ATP/ADP exchange was mediated by ANT1 (b). The calculated ATP–ADP exchange rates were the slopes of the regression lines of the data in (a). Values represent mean ± SE; *n* > 5. *p < 0.05 by Student's *t*‐test. (c). Knockdown of *ANT1* in YD2 cells brought the accelerated ATP–ADP exchange rate back to normal. Values represent mean ± SE; *n* = 14. *p < 0.01 by Student's *t*‐test. Western blot was performed to confirm the knockdown effect of pSuper‐siANT1. RCAN1.1S protein was detected with 9E10 (anti‐myc) antibody and endogenous ANT1 was detected with anti‐ANT1 antibody. Superoxide dismutase 2 (SOD2) was used as mitochondrial loading control. (d). Over‐expression of *RCAN1.1* and *ANT1* resulted in more Ca^2+^‐induced mPTP opening. Assay of mPTP opening was determined in SH‐SY5Y, YD2, SH‐SY5Y transfected with RCAN1.1 and ANT1 expression vectors. mPTP opening was indicated as a decrease in the initial calcein fluorescence (fluo.). The cells were treated with 5 μM ionomycin, 5 μM bongkrekic acid (BKA), 1 μM carboxyatractyloside (CATR), 5 μM ionomycin + 5 μM BKA, 5 μM ionomycin + 1 μM CATR, respectively. Values represent mean ± SE; *n* = 5. *p < 0.05 by Student's *t*‐test. (e). Knockdown of *ANT1* in YD2 cells reduced the Ca^2+^‐induced mPTP opening. The increase of calcein fluorescence indicated the reduction of mPTP opening. Values represent mean ± SE; *n* = 6. *p < 0.05 by Student's *t*‐test.

The fundamental function of ANT1 is the exchange function of bringing ADP into the mitochondrial matrix and bringing ATP out to the cytosol (Dahout‐Gonzalez *et al*. 2006). To find out whether ANT1 function was altered by RCAN1, we measured the ATP–ADP exchange rate in the digitonin‐permeabilized cells. Data displayed that the ATP–ADP exchange rate (nmol/s) was accelerated from 10.52 ± 0.56 nmol/s in SH‐SY5Y cells to 12.79 ± 0.75 nmol/s in YD2 cells (*p* < 0.05, Fig. 4b, lane 1 vs. lane 2), and from 10.83 ± 1.04 nmol/s in control cells to 15.55 ± 1.87 nmol/s in *RCAN1.1S* transiently transfected SH‐SY5Y cells (*p* < 0.05, Fig. 4b, lane 3 vs. lane 4), and ANT1 over‐expression increased the ATP–ADP exchange rate from 10.43 ± 1.04 nmol/s in control cells to 14.57 ± 1.21 nmol/s (*p* < 0.05, Fig. 4b, lane 5 vs. lane 6). To further verify whether ANT1 mediated the effect of RCAN1 on ATP–ADP exchange, we knocked down the elevated *ANT1* expression in YD2 cells using ANT1 shRNA vector. The ATP–ADP exchange rate was decreased from 13.14 ± 0.60 nmol/s to 9.44 ± 0.84 nmol/s (*p* < 0.01, Fig. 4c, black vs. gray), corresponding with decreased protein expression of ANT1. These data suggested that RCAN1 had an effect on ATP/ADP exchange rate via its interaction with ANT1.

### RCAN1.1S affected Ca^2+^‐induced mPTP opening via ANT1

In addition to ATP–ADP exchange, ANT1 also plays a central role in modulating the sensitivity of mPTP to Ca^**2+**^ (Kokoszka *et al*. 2004; Dahout‐Gonzalez *et al*. 2006; Leung and Halestrap 2008). CATR stabilizes the c‐conformation of the ANT1 and sensitizes the mPTP to Ca^**2+**^, while BKA stabilizes the m‐conformation of the ANT1 and makes the mPTP insensitive to Ca^**2+**^ (Dahout‐Gonzalez *et al*. 2006). To further examine whether *RCAN1.1S* over‐expression affected mPTP opening through its up‐regulation on ANT1, mPTP opening was measured by a calcein fluorescence decrease. Though the basal level of calcein fluorescence showed no difference (Fig. 4d, lane 1–6), under the treatment of 5 μM Ca^**2+**^ ionophore ionomycin that triggered mPTP opening, the calcein fluorescence was further decreased by RCAN1.1S or ANT1 over‐expression (Fig. 4d, lane 7–12). The treatment of BKA alone had no influence on the basal level of calcein (Fig. 4d, lane 13–18), while treatment of CATR alone decreased the calcein fluorescence via inducing mPTP opening, and the fluorescence was also further decreased by RCAN1.1S or ANT1 over‐expression (Fig. 4d, lane 19–24). The effect of RCAN1.1S on mPTP opening was abolished by BKA (Fig. 4d, lane 25–30) and intensified by CATR (Fig. 4d, lane 31–36). Furthermore, knockdown of ANT1 by shRNA vector in YD2 cells brought back the mPTP opening induced by RCAN1.1S (Fig. 4e, lane 6, 12, 18 compared to lane 5, 11, 17, respectively). The data here demonstrated that RCAN1 affected mPTP opening through ANT1.

### RCAN1.1S exacerbated Ca^2+^‐induced mitochondrial swelling and compromised Ca^2+^ retention capacity via ANT1

**Figure 5:**
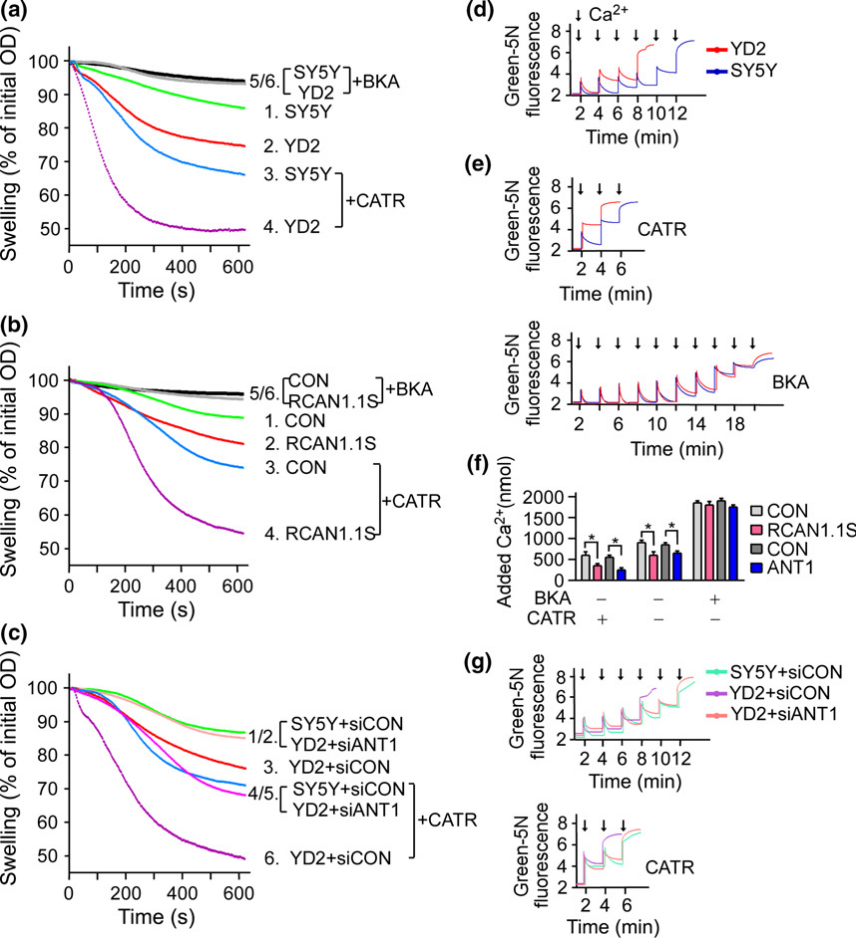
Over‐expression of *RCAN1.1S* exacerbated Ca^2+^‐induced mitochondrial swelling and compromised Ca^2+^ retention capacity. (a) Over‐expression of *RCAN1.1S* exacerbated Ca^2+^‐induced mitochondrial swelling. Ca^2+^‐induced mitochondrial swelling of SH‐SY5Y and YD2 cells in the absence (1.2.) and presence of carboxyatractyloside (CATR) (3.4.) or bongkrekic acid (BKA) (5.6.), expressed as a percentage decrease in the initial absorbance of 540 nm. (b) Ca^2+^‐induced mitochondrial swelling was measured in primary rat neurons infected with *RCAN1.1S* expression lentivirus. (c) Knockdown of adenine nucleotide translocator (*ANT1*) in YD2 cells abolished the effect of RCAN1 on Ca^2+^‐induced mitochondrial swelling. (d and e). Over‐expression of *RCAN1.1S* in YD2 cells compromised Ca^2+^ retention capacity. Extra‐mitochondrial Ca^2+^ was measured fluorometrically with the calcium indicator Calcium green‐5N in the absence (d) and presence (e) of ANT1 ligands CATR and BKA. Traces of Ca^2+^ retention by isolated mitochondria from YD2 (red line) and SH‐SY5Y cells (blue line) were shown. The total amount of Ca^2+^ added until mitochondrial permeability transition pore (mPTP) opening indicated by a double increase above the baseline fluorescence was interpreted as Ca^2+^ retention capacity. (f) Over‐expression of *RCAN1.1S* and *ANT1* compromised the calcium retention capacity of primary rat neurons. Neurons were infected with lentivirus expressing RCAN1.1S and ANT1, respectively. Y axis indicated the total amount of Ca^2+^ added until mPTP opening. (g). Knockdown of *ANT1* in YD2 cells abolished the effect of RCAN1.1S on Ca^2+^ retention capacity. Values represent mean ± SE; *n* = 4. *p < 0.05 by Student's *t*‐test.

mPTP opening leads to a series of consequences, including Ca^**2+**^ retention incapacity, massive swelling of mitochondria, rupture of the outer membrane, release of Cyt *c* or the apoptosis‐inducing factor, and eventually cell death (Halestrap *et al*. 2002; Schwarz *et al*. 2007; Tsujimoto and Shimizu 2007; Leung and Halestrap 2008). Mitochondrial swelling caused by influx of solutes across the inner membrane could be detected by measuring a decrease in the absorbance at 540 nm. Mitochondria Ca^2+^ retention capacity was reflected by the total Ca^**2+**^ injection pulses in the reaction before mPTP opening. The OD540 showed that YD2 cells had a larger degree of swelling than SH‐SY5Y cells (Fig. 5a, curve 2 vs. curve 1 *p* < 0.05). The exacerbation of mitochondrial swelling was abolished by BKA and further amplified by CATR. Similar results were observed in rat primary neurons infected with *RCAN1.1S* expression lentivirus (Fig. 5b). Also, the difference can be abolished by the knockdown of ANT1 with shRNA vector (Fig. 5c). In addition, RCAN1.1S expression reduced Ca^2+^ retention capacity (Fig. 5d, *p* < 0.05). And, the effect on Ca^2+^ retention capacity by RCAN1.1S was abolished by BKA and further amplified by CATR (Fig. 5e). Knockdown of ANT1 in YD2 cells brought back the effect on calcium retention capacity (Fig. 5g). Furthermore, similar results were obtained in metabolically active mitochondria from cultured primary rat neurons infected with lentivirus expressing *RCAN1.1S* and *ANT1* (Fig. 5f). The data here demonstrated that RCAN1 affect mitochondrial swelling and calcium retention capacity via its interaction with ANT1.

### RCAN1.1S induced Cyt *c* release and cell apoptosis via ANT1

**Figure 6:**
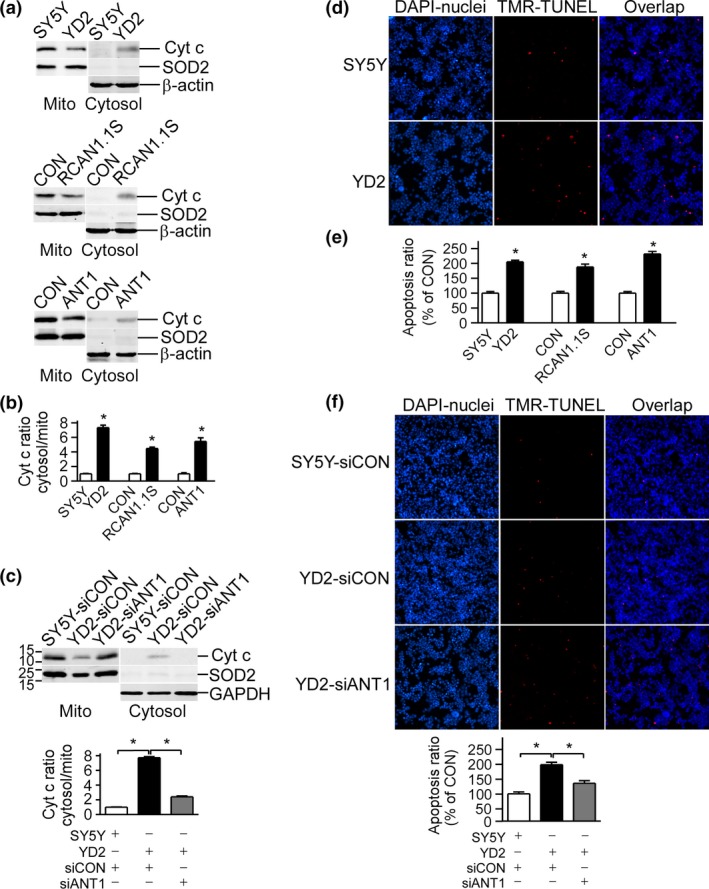
Over‐expression of *RCAN1.1S* facilitated cytochrome *c* (Cyt *c*) release and cell apoptosis, mediated by increased adenine nucleotide translocator (ANT1). (a) Over‐expression of *RCAN1.1S* and *ANT1* increased release of Cyt *c* from mitochondria to cytosol. Mitochondria and cytosol fractions were isolated from SH‐SY5Y, YD2 (top blots of a), and SH‐SY5Y cells transiently transfected with RCAN1.1S (middle blots of a) and ANT1 (bottom blots of a). The cell lysates were separated on a 16% Tris‐tricine sodium dodecyl sulfate–polyacrylamide gel electrophoresis gel. Cyt c was detected by the anti‐Cyt c antibody. Anti‐superoxide dismutase 2 (SOD2) mAb was used to detect SOD2 in mitochondria. (b) Ratio of Cyt c in cytosol to mitochondria of (a). Cyt c was normalized to GAPDH in cytosol fraction and normalized with SOD2 in mitochondria fraction. Values represent mean ± SE; *n* = 3. *p < 0.05 by Student's *t*‐test. (c) Knockdown of *ANT1* in YD2 cells reduced the release of Cyt c. Values represent mean ± SE; *n* = 3. *p < 0.01 by Student's *t*‐test (d). Over‐expression of *RCAN1.1S* in YD2 cells induced cell apoptosis. Apoptotic cells were measured by TUNEL staining. Nuclei were counter‐stained with DAPI. Apoptotic cells were indicated by a magenta color, which corresponded to an overlap of red TUNEL and blue DAPI staining. The results were analyzed by a Leica fluorescent microscope. Scale bars: 100 μm (e). Relative apoptosis ratio of SH‐SY5Y, YD2, and SH‐SY5Y cells transiently transfected with RCAN1.1S and ANT1 expression vectors. Values represent mean ± SE; *n* = 4. *p < 0.05 by Student's *t*‐test. (f). Knockdown of *ANT1* in YD2 cells reduced apoptosis. Scale bars: 100 μm. Values represent mean ± SE; *n* = 6. *p < 0.05 by Student's *t*‐test.

To further verify whether mPTP opening induced by RCAN1 would result in Cyt *c* release and cell apoptosis, we examined the translocation of Cyt *c* from mitochondria to cytosol using western blot. A decrease of Cyt *c* in mitochondria and an increase of Cyt *c* in cytosol were observed in SH‐SY5Y cells stably or transiently over‐expressing RCAN1.1S (Fig. 6a and b). *ANT1* over‐expression displayed similar pattern of Cyt *c* release from mitochondria to cytosol (lane 5 and 6 of Fig 6b), while knockdown of *ANT1* using shRNA vector abolished the translocation of Cyt *c* induced by RCAN1.1S (Fig. 6c). To further investigate the outcome of Cyt *c* release, TUNEL was used to monitor cell apoptosis. Consistent with our previous reports (Sun *et al*. 2011), TUNEL assay showed more cell apoptosis in cells over‐expressing RCAN1.1S (lane 1–4 of Fig. 6e). Similar results were obtained with ANT1 over‐expression (lane 5 and 6 of Fig. 6e), and ANT1 knockdown protected cells from RCAN1.1S induced apoptosis (Fig. 6f). The data here suggested that RCAN1 induced Cyt *c* release and neuronal cell apoptosis through mPTP opening via its interaction with ANT1.

## Discussion

Our study here showed that RCAN1.1S elevated *ANT1* expression, which resulted in mitochondrial dysfunctions including ATP/ADP exchange rate, mPTP opening, mitochondrial swelling, and Cyt *c* release. Furthermore, knockdown of ANT1 in cells over‐expressing RCAN1.1 brought back these perturbations to normal. The study suggested that ANT1 and mitochondrial perturbations contributed to the cell apoptosis induced by *RCAN1* over‐expression. Our previous study has shown that *RCAN1* over‐expression in primary neurons induced neuronal apoptosis, mediated by Cyt *c* release, and activation of caspase 9 and caspase 3 (Sun *et al*. 2011). The study here further provided the molecular mechanism which RCAN1 interacted with ANT1 in mitochondria and facilitated mPTP opening, resulting in Cyt *c* release and apoptosis.

RCAN1 can physically interact with calcineurin subunit A through its C‐terminus aa 141–197 and inhibit calcineurin‐NFAT signaling. The effect of RCAN1.1S on ANT1 is independent of its effect on calcineurin‐NFAT since ANT1 did not respond to calcineurin inhibitor FK506. And, the RCAN1 C‐terminus that inhibited calcineurin‐NFAT signaling did not affect ANT1 expression. Furthermore, we identified that the aa 1–103 of RCAN1.1S was responsible for its effect on ANT1 in mitochondria. RCAN1.4 isoform has similar effect on ANT1 with RCAN1.1S isoform. We also had data showing that RCAN1.1L isoform had similar effect on ANT1 in mitochondria. Our data suggested that the effect of RCAN1 on ANT1 in mitochondria is a common function of RCAN1 isoforms and independent of its inhibition on calcineurin.

The abundance of mRNA in the cell is a function of not only its synthesis, processing, and nuclear export rate but also of its degradation rate in the cytoplasm. mRNA degradation rates often change in response to stimulus. RNA binding proteins or non‐coding RNAs bind to their cis‐acting elements in mRNA and affect the mRNA degradation rates via their ability to recruit or exclude mRNA degradation machinery. The 28–103 aa of RCAN1.1S, a common domain among the isoforms RCAN1.1S, RCAN1.4, and RCAN1.1L, was predicted to be an RNA binding domain by RCSB PDB database. It would be of great interest to examine whether this RNA binding activity of RCAN1 would contribute to the stability of ANT1 mRNA in the future.

*ANT1* missense mutations have been found in autosomal dominant progressive external ophthalmoplegia with mitochondrial DNA deletions‐2 (Kaukonen *et al*. 2000). Senger's syndrome and Senger's‐like syndrome are also associated with severe depletion of ANT1 protein and absence of ANT1 function, but no *ANT1* gene abnormality has been identified (Jordens *et al*. 2002; Morava *et al*. 2004). It would be interesting to investigate whether RCAN1 played some role in regulating the ANT1 protein expression in these diseases.

## Supporting information


**Figure S1.** RCAN1 larger isoform increased endogenous ANT1 expression.
