# Supplementary material for: The regulator of calcineurin 1 increases adenine nucleotide translocator 1 and leads to mitochondrial dysfunctions
Source: J Neurochem. 2016 Dec 20;140(2):307–19. doi: 10.1111/jnc.13900 (PMC5248620; doi:10.1111/jnc.13900)
Supplement: Supplementary file 1 — Figure S1. RCAN1 larger isoform increased endogenous ANT1 expression. [file JNC-140-307-s001.pdf]

**The regulator of calcineurin 1 increases adenine nucleotide translocator 1 and leads to mitochondrial dysfunctions**

Hui Jiang[1,4*], Chen Zhang[1*], Yu Tang[1], Juan Zhao[1], Tan Wang[3], Heng Liu[1], Xiulian Sun[2†]

From [1] Otolaryngology Key Lab, [2] Brain Research Institute, [3] Department of Geriatrics, Qilu Hospital of Shandong University, No.107 West Wenhua Road, Jinan 250012, Shandong Province, China;

[4] Department of Pediatrics, 2nd Hospital of Shandong University, No. 44 West Wenhua Road, Jinan 250011, Shandong Province, China;

*These authors contributed equally to this work.

† To whom correspondence should be addressed: Brain Research Institute, Qilu Hospital of Shandong University, No.107 West Wenhua Road, Jinan 250012, Shandong Province, China; Tel/Fax: (86)53182169284; Email: xiulians@gmail.com.

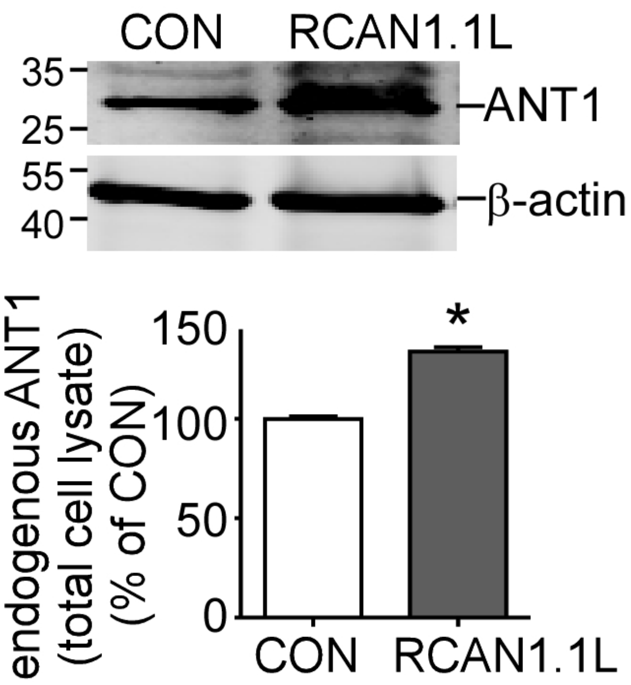

Supplementary Fig. 1: RCAN1 larger isoform increased endogenous ANT1 expression. SH-SY5Y cells were transfected with RCAN1.1L expression vector pRCAN1.1Lmyc (Wu & Song 2013). 48 hours after transfection, cells were harvested and lysed with RIPA lysis buffer. The whole cell lysates were separated with 12% SDS-PAGE gel. Endogenous ANT1 was detected with anti-ANT1 antibody (ab110322, abcam, USA). β-actin was used as loading control. N=4, * p=0.0005.

Reference：

Wu, Y. and Song, W. (2013) Regulation of RCAN1 translation and its role in oxidative stress-induced apoptosis. *FASEB journal : official publication of the Federation of American Societies for Experimental Biology,* **27,** 208-221.
